# Miescher’s Granulomatous Cheilitis: A Rare Cause of Recurrent Lip Inflammation

**DOI:** 10.7759/cureus.113750

**Published:** 2026-07-31

**Authors:** Valeria Gonzalez Quiroz, Sergio Eduardo Arroyo Jaramillo, Arturo Soto Ontiveros, Juan Lorenzo Perez Castro, Pablo Gabriel Trujillo Caraveo, Onfalia Baez Perez, Daniel Alejandro Oliva Perez

**Affiliations:** 1 Internal Medicine, Mexican Social Security Institute, Durango, MEX; 2 Dermatology, Mexican Social Security Institute, Durango, MEX

**Keywords:** cheilitis granulomatosa miescher, granulomatous disorder, lower lip, methyl prednisolone, noncaseating granulomas

## Abstract

Miescher’s granulomatous cheilitis (GC) is a rare, noninfectious, chronic, idiopathic granulomatous disorder characterized by recurrent swelling of the lips. It is considered part of the triad of Melkersson-Rosenthal syndrome, a neurological and cutaneous disorder characterized by orofacial edema, peripheral facial nerve palsy, and a fissured tongue. However, the complete triad is uncommon, and Miescher’s GC may present as a monosymptomatic form. Due to its rarity, the condition has a broad differential diagnosis and poses a diagnostic challenge. We report the case of a 33-year-old woman with no relevant medical history who presented with chronic, recurrent swelling of the lower lip. She was initially treated for a presumed allergic reaction with antihistamines, without improvement. A lip biopsy was performed, revealing histopathological findings consistent with GC. The patient was treated with prednisone and doxycycline, followed by intralesional methylprednisolone injections, resulting in a favorable clinical response without recurrence. This case highlights the importance of recognizing localized orofacial manifestations and providing timely, individualized treatment to reduce the risk of recurrence.

## Introduction

Granulomatous cheilitis (GC) is a rare, chronic, idiopathic disorder characterized by persistent swelling of one or both lips resulting from noncaseating granulomatous inflammation. It belongs to the clinical spectrum of orofacial granulomatosis and represents the most common monosymptomatic variant of Melkersson-Rosenthal syndrome, a condition classically defined by the triad of recurrent orofacial edema, peripheral facial nerve palsy, and a fissured tongue [1]. Although its precise etiology remains elusive, it is considered multifactorial, involving genetic susceptibility, immune dysregulation, delayed hypersensitivity reactions, and potential infectious triggers. Furthermore, GC has been associated with systemic granulomatous disorders, most notably Crohn's disease and sarcoidosis; hence, these conditions must be systematically excluded during the diagnostic workup [1-2].

Diagnosis is frequently delayed as initial episodes often mimic more common conditions, including angioedema, contact dermatitis, or localized infectious diseases. Consequently, a histopathological examination demonstrating noncaseating granulomas, combined with rigorous clinicopathological correlation and the exclusion of systemic mimics, remains the cornerstone of diagnosis [3]. To date, standardized therapeutic guidelines have not been established, and corticosteroids, particularly intralesional injections, remain the mainstay of management. Herein, we present a case of isolated Miescher's GC successfully managed with systemic corticosteroids followed by intralesional methylprednisolone, illustrating the classic clinical trajectory and therapeutic challenges of this condition [4].

## Case presentation

**Figure 1:**
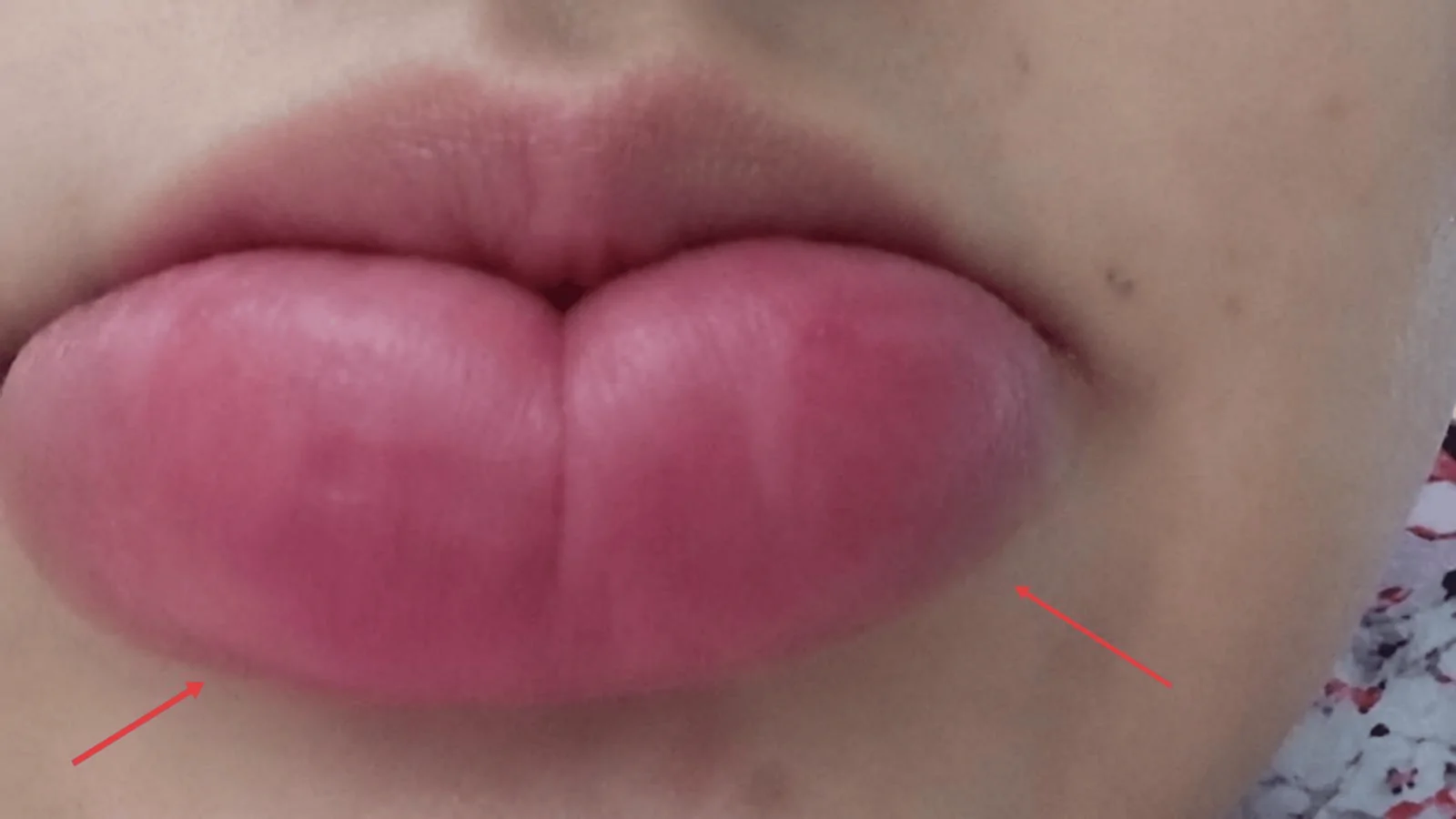
Initial clinical presentation with predominance of the lower lip Predominant involvement of the lower lip. The lower lip appears enlarged, with a smooth, slightly erythematous surface, and increased volume extending along the vermilion border. The red arrows indicate the areas of maximum edema and swelling affecting both lower lips and the adjacent perioral soft tissue

**Figure 2:**
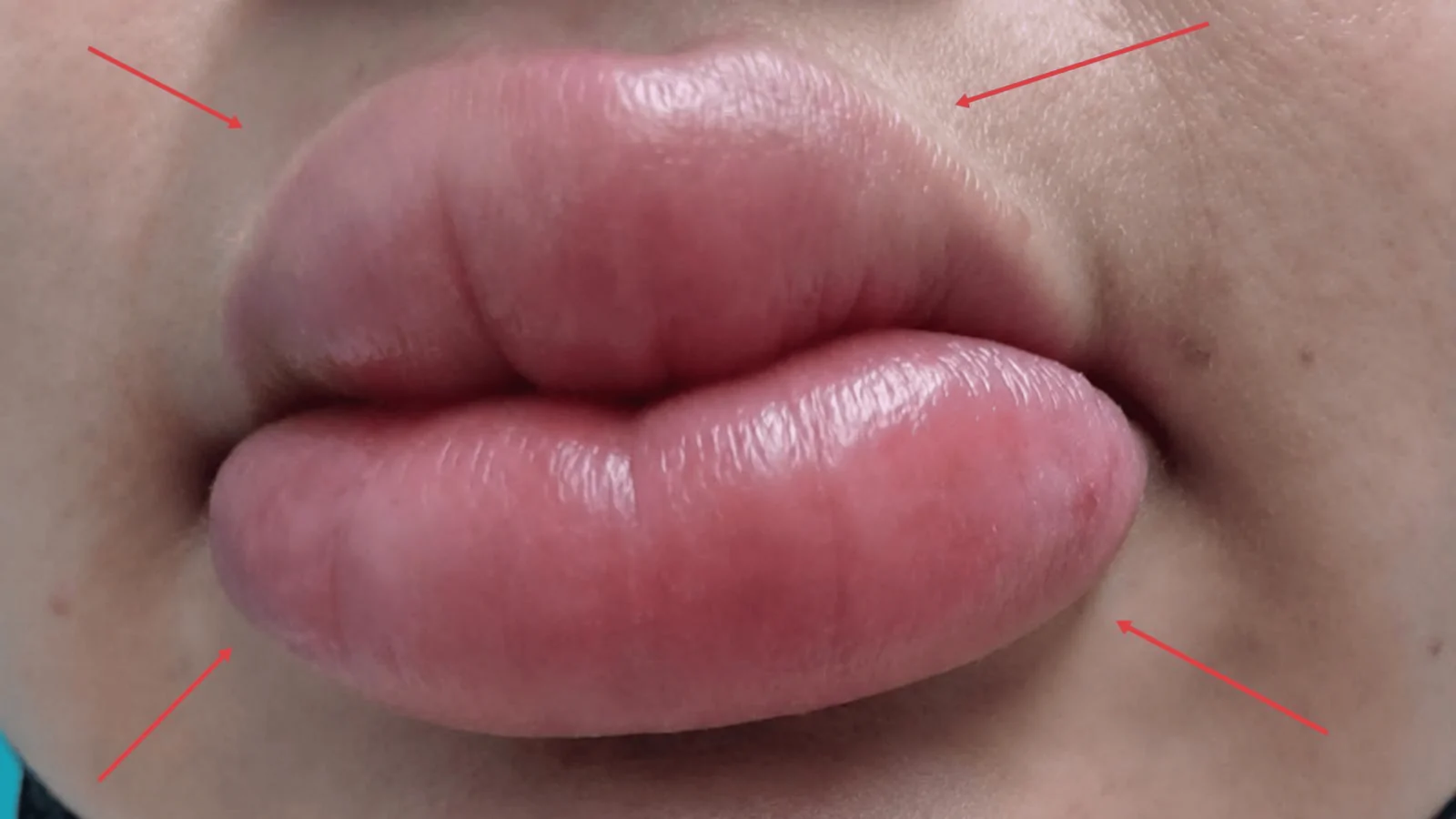
Clinical progression two weeks later demonstrating diffuse bilateral lip edema, more pronounced in the lower lip The lower lip shows marked, diffuse, smooth swelling with increased volume and mild erythematous discoloration, extending beyond the vermilion border into the adjacent perioral soft tissues. The upper lip also shows mild-to-moderate diffuse edema. The red arrows indicate the areas of swelling and edema of the perioral soft tissues surrounding the upper and lower lips

A 33-year-old woman with an unremarkable medical history presented with a three-year history of recurrent, painless, nonpruritic lip swelling, predominantly affecting the lower lip. She denied any respiratory, gastrointestinal, or neurological symptoms, and physical examination confirmed the absence of facial nerve paralysis or a fissured tongue. Localized examination revealed a soft edema in the midportion of the lower lip accompanied by two small superficial fissures (Figure 1). No other cutaneous or mucosal lesions were detected (Figure 2).

**Figure 3:**
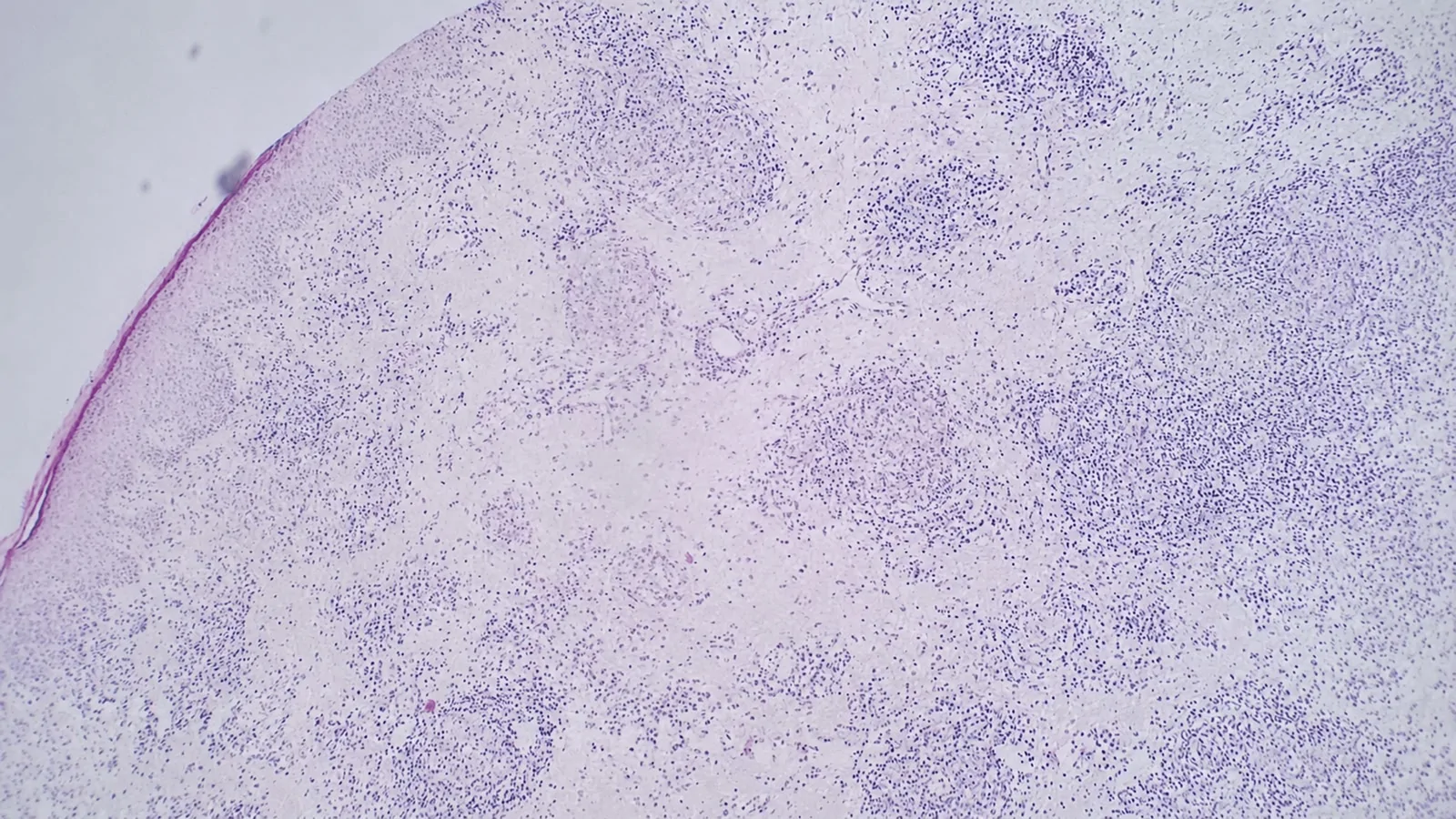
Lip biopsy stained with hematoxylin and eosin (40×) Multiple well-formed, noncaseating epithelioid granulomas are observed in the lamina propria and submucosa, accompanied by a lymphocytic inflammatory infiltrate and mild stromal fibrosis. No central necrosis is identified

**Figure 4:**
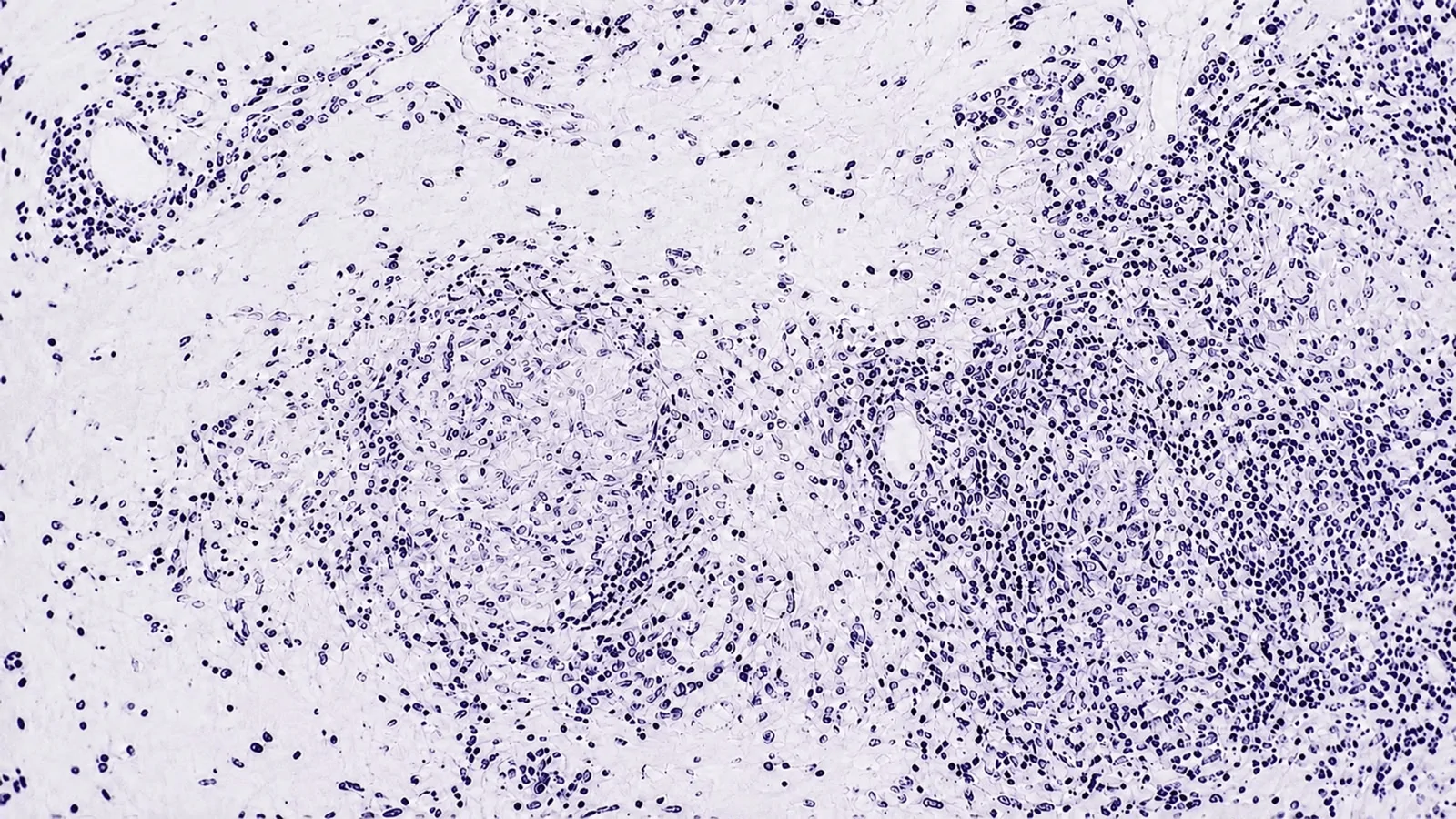
Lip biopsy stained with hematoxylin and eosin (100×) Lip biopsy with noncaseating granulomas composed of epithelioid histiocytes with a perigranulomatous lymphocytic infiltrate

The patient had been managed initially for a suspected allergic reaction with systemic antihistamines, yielding no clinical improvement. Upon dermatological evaluation, a comprehensive diagnostic workup was initiated, including a complete blood count, blood chemistry panel, liver function tests, serum angiotensin-converting enzyme (ACE) levels, and viral serologies (Table 1). A punch biopsy of the lower lip was performed. Histopathological examination with hematoxylin and eosin (H&E) staining of a lip biopsy, including mucosa and submucosal tissue, demonstrated multiple well-formed epithelioid granulomas, without caseation, located predominantly in the superficial and deep lamina propria and submucosa. The granulomas were surrounded by a mild-to-moderate lymphocytic inflammatory infiltrate and were associated with interstitial fibrosis and stromal edema. No central necrosis was identified. Occasionally, multinucleated giant cells were observed within some granulomas. No significant vasculitis or perineural inflammation was observed. Polarized light examination did not reveal birefringent foreign material. Periodic acid-Schiff (PAS) staining was negative for fungal organisms. Grocott methenamine silver (GMS) staining and microbiological cultures were not performed due to the absence of clinical or histopathological suspicion of deep fungal infection. Molecular microbiological studies were not considered clinically indicated. The overall findings were consistent with GC after clinicopathological correlation (Figures 3-4). Further systemic evaluation, including a chest X-ray and a QuantiFERON-TB Gold test, was unremarkable, effectively ruling out sarcoidosis and tuberculosis. Although the patient denied gastrointestinal symptoms, fecal calprotectin levels were also measured and found to be within normal limits.

**Table 1:** Laboratory tests HBV: hepatitis B virus; HCV: hepatitis C virus; HIV: human immunodeficiency virus; VDRL: Venereal Disease Research Laboratory; ACE: angiotensin-converting enzyme

Variables	Results		Units	Normal parameters
Leukocytes	6.82		X10^13^/L	(4-11)
Erythrocytes	4.69		X10^12^/L	(4-5)
Hemoglobin	14		g/dL	(12-17)
Hematocrit	42		%	(35-52)
Mean corpuscular volume	85		fL	(80-100)
Mean corpuscular hemoglobin	31		pg/cell	(27-31.2)
Platelets	356		X 10^3^/mm^3^	(130-400)
Neutrophils	3.86		X10^3^/mm^3^	(2-8)
Lymphocytes	2.08		X10^3^/mm^3^	(1-5)
Eosinophils	0.10		X10^3^/mm^3^	(0-0.40)
Basophils	0.10		X10^3^/mm^3^	(0-0.20)
Glucose	80		mg/dl	(74-106)
Urea	20		mg/dl	(19-43)
Urea nitrogen	10		mg/dl	(9.0-20)
Creatinine	0.6		mg/dl	(0.60-1.20)
Total proteins	7.1		g/dL	(6.3-8.2)
Albumin	3.5		g/dL	(3.5-5)
Total bilirubin	0.6		mg/dl	(0.2-1.3)
Indirect bilirubin	0.4		mg/dl	(0-1.1)
Direct bilirubin	0.2		mg/dl	(0-1)
Aspartate aminotransferase	17		U/l	(14-36)
Alanine aminotransferase	20		U/l	(< 35)
Alkaline phosphatase	100		U/l	(38-126)
Lactate dehydrogenase	127		U/l	(120-246)
Sodium	138		mmol/l	(137-145)
Potassium	4.4		mmol/l	(3.5-5.1)
Serum chloride	106		mmol/l	(98-107)
Calcium	8.5		mg/dl	(8.4-10.2)
Phosphorus	4		mg/dl	(2.5-4.5)
Magnesium	1.6		mg/dl	(1.6-2.4)
Prothrombin time	15		Seconds	(11.9-15.6)
Prothrombin time witness	13.5		Seconds	
International standardized index	1.21			
Partial thromboplastin time	36		Seconds	(32.22-40.14)
Partial thromboplastin time index	36.18		Seconds	
HBV surface antigen		Nonreactive	Nonreactive	Negative
Anti-HCV antibody		Nonreactive	Nonreactive	Negative
Anti-HIV antibody		Nonreactive	Nonreactive	Negative
VDRL		Nonreactive	Nonreactive	Negative
ACE		28	UI/L	(8-53)
QuantiFERON-TB Gold		<0.10	UI/L	(<0.35)

**Figure 5:**
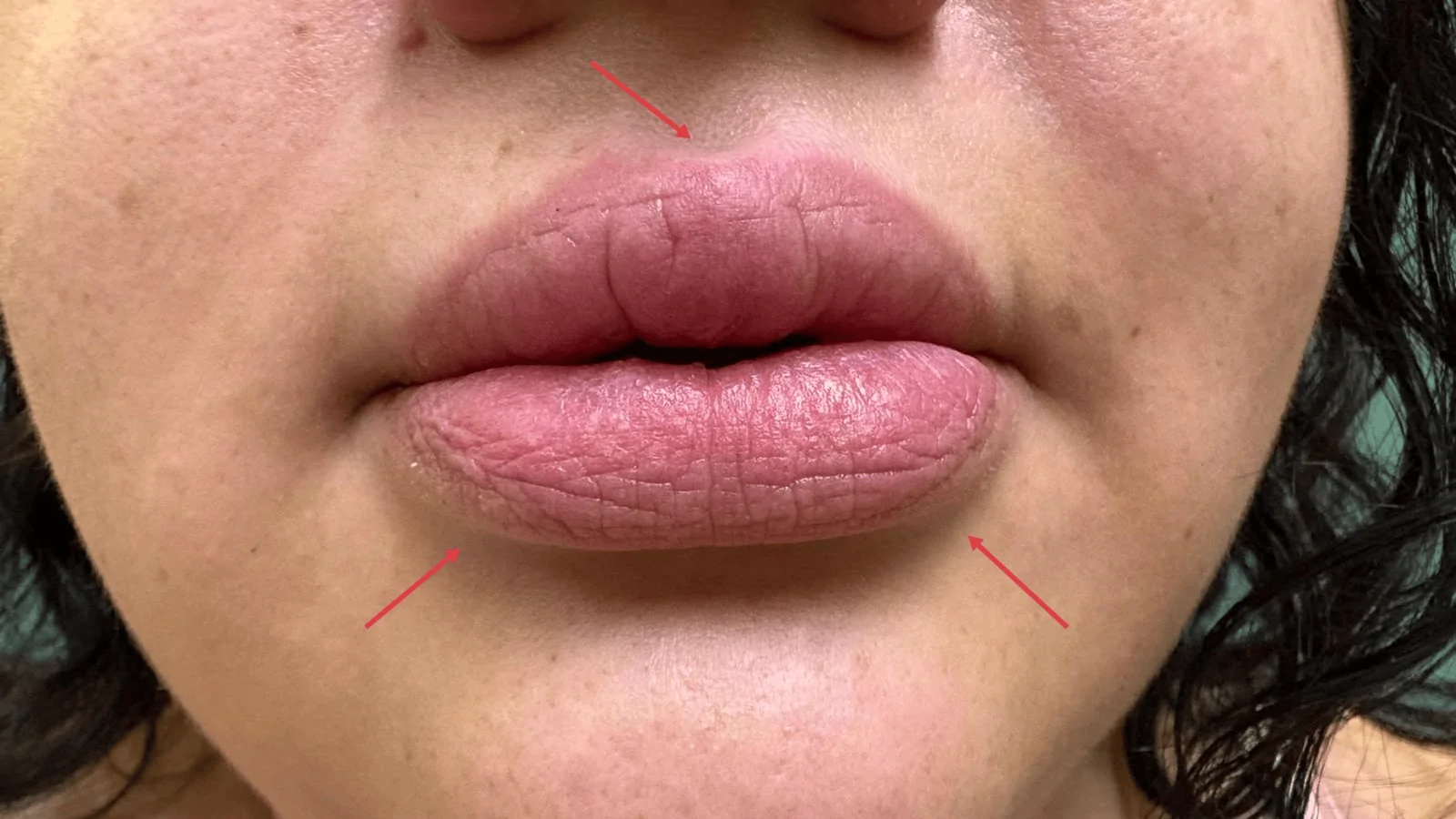
Frontal clinical photograph obtained during the resolution phase of granulomatous cheilitis The upper and lower lips show marked improvement in the previously documented edema, with the vermilion border architecture preserved and an almost normal lip contour. A slight residual prominence of the lower lip and subtle dryness of the vermilion border are observed, with no evidence of active inflammation, ulceration, crusting, or fissures

Treatment was initiated with systemic steroids: oral prednisone 50 mg/day for seven days and doxycycline 100 mg/day for two months without improvement. Subsequently, the patient received three sessions of intralesional methylprednisolone acetate (40 mg/mL), with a total volume of 0.5 mL per session, administered at four-week intervals. The procedure was well tolerated, and no local adverse effects were observed. At the 12-week follow-up, counted from the start of intralesional therapy, almost complete clinical remission was achieved, with an approximate 90% improvement (Figure 5). No recurrence was observed at the six-month follow-up.

## Discussion

Miescher's GC is a rare, chronic inflammatory condition characterized by recurrent or persistent edema of one or both lips and, histologically, non-caseating granulomatous inflammation. It is considered part of the orofacial granulomatosis spectrum and represents the most frequent monosymptomatic form of Melkersson-Rosenthal syndrome [5].

The epidemiology of this condition is unclear; its prevalence has been estimated at approximately 0.08% in the general population, although underreporting is likely due to delayed diagnosis. It can occur at any age, but is more common in young adults, with a slight female predominance. In Mexico, there are no epidemiological studies; the available information is limited to case reports and small clinical series [6].

The epidemiology of this condition is unclear, although underreporting is likely due to delayed diagnosis. It can occur at any age, but is more common in young adults, with a slight female predominance. In Mexico, there are no epidemiological studies; the available information is limited to case reports and small clinical series [6].

From a pathophysiological standpoint, the characteristic finding is the presence of noncaseating granulomas in the lamina propria and submucosal tissue, frequently accompanied by perivascular lymphocytic infiltrate, edema, and impaired lymphatic drainage. Chronic inflammation can lead to progressive lymphatic obstruction, favoring recurrent inflammation and, in advanced stages, fibrosis of the lip tissue [7].

Diagnosis is complex due to the lack of clear criteria. In early stages, recurrent edema is frequently attributed to allergic processes. In this case, the patient initially received antihistamines for suspected angioedema, without clinical improvement. Due to persistent and recurrent inflammation of the lower lip, a biopsy was performed, which confirmed GC [8].

The differential diagnosis is broad and primarily includes angioedema, Crohn's disease, sarcoidosis, tuberculosis, fungal infections, and granulomatosis with polyangiitis. Systemic and intralesional corticosteroids form the basis of treatment [9]. Current evidence supports the use of corticosteroids, particularly intralesional corticosteroids, as first-line treatment [10-11]. In this case, a short course of systemic prednisone followed by high-dose intralesional therapy combined with doxycycline was chosen to achieve rapid anti-inflammatory effects and minimize long-term steroid toxicity. However, given that treatment recommendations are based primarily on case reports, case series, and retrospective data, and recurrence remains frequent [10-11], a longer follow-up period, exceeding six months, is warranted to definitively establish the long-term durability of this specific regimen.

This case underscores the importance of considering GC in patients with recurrent lip edema unresponsive to antihistamines or other treatments. It also highlights the value of histopathological evaluation for diagnostic confirmation and exclusion of systemic granulomatous diseases.

## Conclusions

This case highlights the typical clinical and histopathological features of GC, a rare inflammatory disorder that remains a diagnostic challenge due to its similarity to other causes of persistent lip swelling. Clinicians should maintain a high index of suspicion in patients with chronic or recurrent lip enlargement that does not respond to conventional treatment, as prompt histopathological evaluation is essential to establish the diagnosis and rule out systemic granulomatous diseases. Given the lack of standardized treatment guidelines, management should be individualized, with rigorous follow-up to monitor therapeutic response and detect potential relapses.
